# Comparison of theoretical proteomes: Identification of COGs with conserved and variable pI within the multimodal pI distribution

**DOI:** 10.1186/1471-2164-6-116

**Published:** 2005-09-09

**Authors:** Soumyadeep Nandi, Nipun Mehra, Andrew M Lynn, Alok Bhattacharya

**Affiliations:** 1 Centre for Computational Biology and Bioinformatics, School of Information Technology, Jawaharlal Nehru University, New Delhi 110067, India; 2 School of Life Sciences, Jawaharlal Nehru University, New Delhi 110067, India

## Abstract

**Background:**

Theoretical proteome analysis, generated by plotting theoretical isoelectric points (pI) against molecular masses of all proteins encoded by the genome show a multimodal distribution for pI. This multimodal distribution is an effect of allowed combinations of the charged amino acids, and not due to evolutionary causes. The variation in this distribution can be correlated to the organisms ecological niche. Contributions to this variation maybe mapped to individual proteins by studying the variation in pI of orthologs across microorganism genomes.

**Results:**

The distribution of ortholog pI values showed trimodal distributions for all prokaryotic genomes analyzed, similar to whole proteome plots. Pairwise analysis of pI variation show that a few COGs are conserved within, but most vary between, the acidic and basic regions of the distribution, while molecular mass is more highly conserved. At the level of functional grouping of orthologs, five groups vary significantly from the population of orthologs, which is attributed to either conservation at the level of sequences or a bias for either positively or negatively charged residues contributing to the function. Individual COGs conserved in both the acidic and basic regions of the trimodal distribution are identified, and orthologs that best represent the variation in levels of the acidic and basic regions are listed.

**Conclusion:**

The analysis of pI distribution by using orthologs provides a basis for resolution of theoretical proteome comparison at the level of individual proteins. Orthologs identified that significantly vary between the major acidic and basic regions maybe used as representative of the variation of the entire proteome.

## Background

A protein's Isoelectric point (pI) – the pH at which a protein has no net charge – is the basis for its isolation using isoelectric focussing and along with Molecular Mass (Mr) is exploited in two dimensional gel electrophoresis used to seperate a cell's protein content. Bjellquist [1,2] has shown that a the pI of a denatured linear protein can be calculated with high accuracy using the pK values of the amino acids responsible for charge. Using these calculations, it is posible to create an image of an organisms theoretical proteome, by plotting the theoretical pI against their theoretical Mr. The distribution of pI in these plots have a multimodal distribution. Early results on bacteria demonstrated a bimodal distribution with peaks centered around pH 5.5 and pH 9 [3]. This bimodality was explained as being caused by the fact that as proteins are least soluble at their pI, they have evolved to have pI's away from neutral pH – which was assumed to be the intracellular pH. Schwartz *et al *[4], further showed the presence of a trimodal pI distribution in Eukaryotes, and observed a correlation of pI to intracellular localisation. Cytoplasmic, nuclear and membrane proteins seemed to lie largely in the acidic, neutral and basic portions of the trimodal distribution, respectively.

Exhaustive work on virtual proteomes has been completed recently by two groups. Weiller *et al *[5] have proved that the multimodal distribution of protein pI is present in randomly generated sequences, and is a function of allowed combinations of amino-acid pKa values, rather than a cause of sequence evolution. A trimodal distribution is present in the virtual proteome of most organisms, with minima at 7.4 and 8.0. Knight *et al *[6] have shown that the variation in proteomes though the trimodal distribution is largely maintained – is influenced by the ecological niche of the organism.

Environmental influences of the proteome are known[7]. Amino acid usage is influenced by the G+C content of a genome [8]. Acidic residues predominate over basic residues in halophilic bacteria [9,10], and compositional properties are further distinguished in thermophilic and mesophilic bacteria – with a preference for salt-bridges (residues with opposite charges) and long-chain hydrophobic residues in the former for increased stability [11].

All studies so far have used the gross properties of the proteome, or broad functional groups (e.g membrane proteins), and not attempted to resolve the multimodal distribution on the basis of individual proteins. This analysis could provide answers to the apparant conflict that though the pI multimodal distribution is caused by the properties of amino acids and not evolutionary factors, environmental influences induce variation in the sizes of each cluster in the distribution. By mapping the variation of pI in orthologs, one can in principal identify proteins whose pI is conserved as well as those whose pI does not seem to be responsible for its function, and whose variation maybe used as markers for an organism's environment.

The COG (Cluster of Orthologous Groups) database [12,13] lists orthologs present across completed genomes. In this study, we consider the subset of an organism's proteome, as specified by the COG database, which contain orthologs present in other genomes – providing a basis to study the variation of pI of individual proteins across genomes.

## Results and discussion

### Comparison of virtual proteomes among different Bacteria organisms

**Figure 1:**
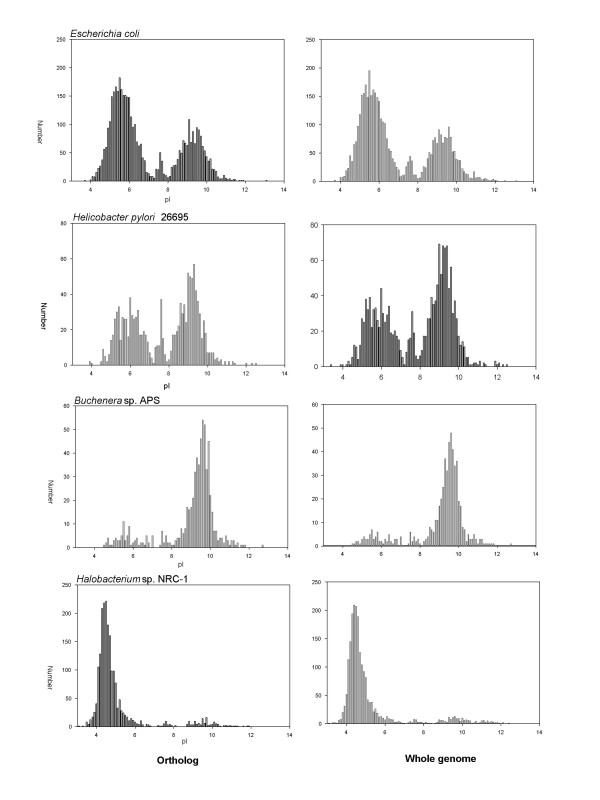
Ortholog (left) and whole genome (right) isoelectric point (pI) frequency distributions for (A) *Escherichia coli *K 12, (B) *Helicobacter pylori *(C) *Buchenera *APS and (D) *Halobacterium *sps NRC-1

**Figure 2:**
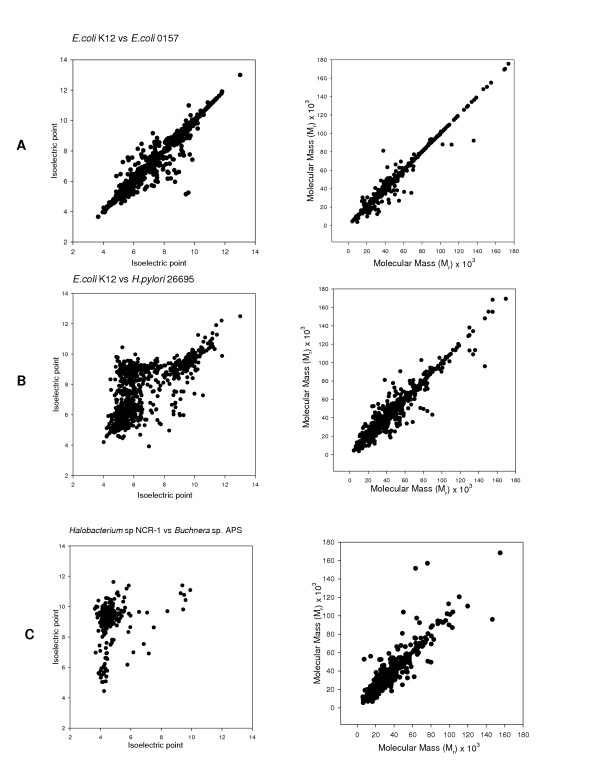
Pairwise comparison of isoelectric point and molecular mass of orthologs. (A) *Escherichia coli *K12 and 0517; (B) *Escherichia coli *K12 and *Helicobacter pylori *(C) *Halobacterium sps *NCR-1 and *Buchnera sps *APS

The predicted proteomes, using values of Mr and pI calculated from the protein ortholog sequence, all displayed a trimodal distribution, with minima at 7.4 and 8.1. For convenience, the three major peaks demarkated by these minima are referred to as the acidic cluster (pI less than 7.4), "neutral" cluster (pI between 7.4 and 8.1) and basic cluster (pI greater than 8.1). This observation of a trimodal distribution is consistent with earlier results calculated from the complete genome [4-6], showing that orthalogs maybe used as representative samples of the complete genome. Figure 1 shows representative plots of proteomes using whole genomes and orthalog sub-sets for *Escherichia coli *K12 and *Helicobacter pylori, Buchenara *and *Halobacterium*. As only orthologs are used, it is possible to compare the pI of two genomes, by using a scatter plot. Diagonal points show invariant pI between the organisms, while a shift in pI is visible as an off-diagonal point. As the trimodal distribution is dominated by the large acidic and basic clusters, A shift in the pI from one cluster to the other is visable on the upper left and lower right quadrants of the plot. Figure 2 shows the pairwise comparison of the pI and Mr for the proteomes of two variants of *E. coli *(K-12 and 0157), between *E. coli *K12 and *H. pylori *which have different environmental pI – and extremophiles *Buchenara *and *Halobacterium*. Closely related variants of the same species do not have much variation in both pI and Mr, however the effect of a changed pH environment – specifically the acidic stomach for *H. pylori *and the basic intestine for *E. coli *– causes a large proportion of orthologs to change pI to compensate for the external pH change. Organisms that have extreme proteome pI distributions are *Halobacterium *– which has a large acidic cluster and *Buchnera *– a large negative cluster. As expected, most orthologs shared by the two organisms show a shift in their pI from one cluster to the other, however a baseline of pI conservation is maintained, implying that the pI maybe a conserved property for a few orthologs. The Mr is much more highly conserved, with the scatter plot clustered along the diagonal.

### Variation in pI among functional categories of COGS

**Figure 3:**
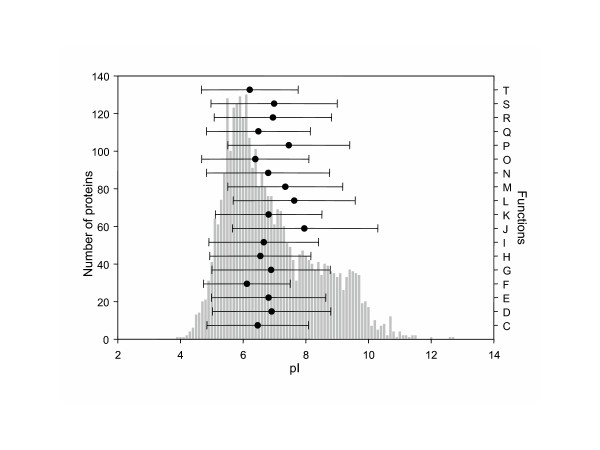
Frequency distribution for COG mean pI (grey vertical bars). The variation of pI (mean and standard deviation) for functionally classified groups of COGs is overlayed. Functional groups denoted by single letters is expanded in Table 1

**Figure 4:**
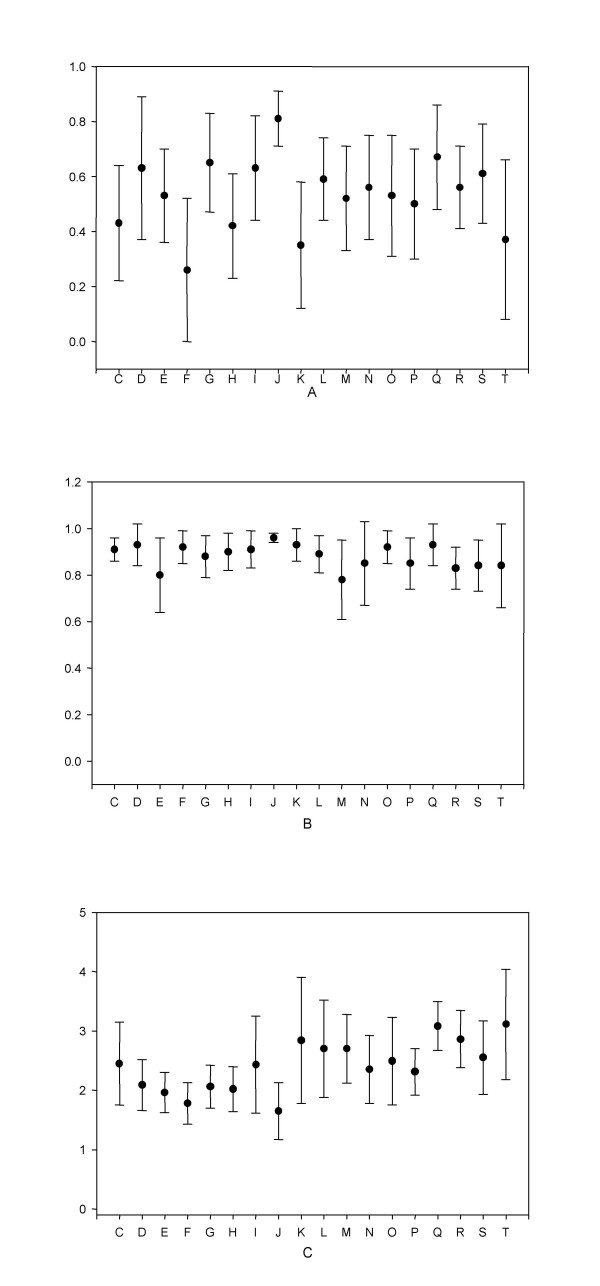
Mean correlation of pI (A), Mr (B) and sequence distance (C) for all organism pairs for the functional groups of COGS. Functional groups are denoted by single letters, expanded in Table 1

The COG database is functional classified into eighteen categories [12] (Table 1, shows a listing of these functions, which the single letter code used in the figures). The distribution of pI values across all organisms for each function, summarised by the mean and standard deviation, is shown in Figure 3. The mean values for each function is close to neutral pH, with a deviation spreading across both clusters. Again the use of orthologs provides the means to correlate conservation of pI between pairs of organisms. The mean pairwise-correlation of ortholog pI corresponding to each function was also computed (Figure 4A), along with the corresponding pairwise correlation of ortholog Mr (Figure 4B). We have used Kruskal-Wallis multiple comparison testing [14] to identify groups that significantly deviate from the expected distribution. Five functional groups deviate significantly, three towards the basic cluster and two towards the acidic cluster (Table 1).

**Table 1:** Variation in pI in functionally classified groups of COGs. The table lists functional groups of COGs as defined in the COG website [22] along with the results of the Dunn Multiple Comparison test. R_i _– mean rank for the group, R_t _– mean rank for sample representing the complete distribution. Significant values are those where |R_t _- R_i_| > 467.35 which is calculated for α = 0.2

		**R**_**i**_	**R**_**i**_** - R**_**t**_
**Metabolism**			
C	Energy production and conversion	2563.72	276.96
E	Amino acid transport and metabolism	2792.99	47.69
**F**	**Nucleotide transport and metabolism**	**2191.12**	**649.56**
G	Carbohydrate transport and metabolism	2958.98	-118.3
H	Coenzyme metabolism	2736.4	104.28
I	Lipid metabolism	2587.87	252.81
Q	Secondary metabolism biosynthesis, transport and catabolism	2479.07	361.62
**Information storage and processing**			
**J**	**Translation, ribosomal structure and biogenesis**	**3399.31**	**-558.63**
K	Transcription	2977.93	-137.25
**L**	**DNA replication, recombination and repair**	**3530.56**	**-689.88**
**Cellular process**			
M	Cell Envelope biogenesis, Outer membrane	3287.13	-446.45
N	Cell motility and secretion	2722.08	118.6
O	Post translation modification, protein turnover, chaperones	2445.01	395.67
**P**	**Inorganic ion transport and metabolism**	**3482.93**	**-642.25**
**T**	**Signal transduction mechanisms**	**2219.23**	**621.45**
D	Cell devision and chromosome partitioning	2836.43	4.25
**Poorly characterized**			
R	General function prediction only	3189.18	-348.5
S	Function unknown	2918.88	-78.2
		R_t _= 2840.68	

Membrane proteins are known to have a preference for the basic cluster, caused by the larger proportion of basic charged residues to compensate for the negatively charged membrane bilayer [4,6]. A functional requirement for either basic or acidic charges could thus influence the protein's pI. Orthologs, by definition, perform the same function in different organisms, and if preference for either basic or acidic charged residues is related to this function, this should be reflected in the protien's pI having a bias for the respective cluster. However, the conservation of pI maybe accidental, especially if the proteins are highly conserved. We have calculated the pairwise distance of all proteins within a COG as a measure of their sequence similarity. The distributions of distance for each functional group is shown in Figure 4C.

Proteins associated with the group "J", involved with translation, ribosomal structure and biogenesis, are dominated by highly conserved ribosomal proteins – and this high level of conservation and intolerance to mutation is reflected in an invariance of pI. This conservation is also reflected in the higher correlation for this group of proteins in their Mr. Other groups which are polarised to either the acidic or basic modes of the pI distribution do not show such a high level of conservation, and pI conservation is dictated by the nature of their function.

### Analysis of pI variation for individual COGS

**Figure 5:**
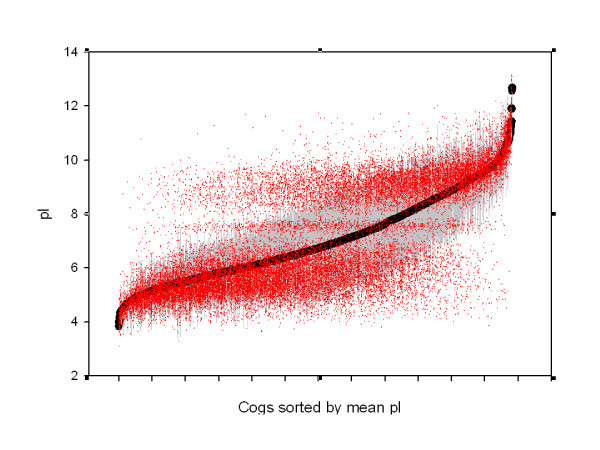
Isoelectric point distribution of orthologs sorted by mean isoelectric point value. Black dot – mean value for COG, grey bar – standard deviation for COG, red point – pI value of individual genes classified under the COG. The sorted list of COG's used for the X-axis is available as an additional file.

The general function of a group of proteins and their level of conservation may dictate a preference for a specific range of pI, as had been shown earlier for membrane proteins, and for functional groups of COGs in the previous section of this paper. Individual proteins maybe identified that have a preference for either the acidic or basic cluster. The scatter plot of the pI of all ortholog proteins used in this analysis is sorted by COG mean pI and plotted in Figure (5). The allowed trimodal regions are clearly visable. However at both the left and right extremes, the scatter plot show that COGs do exist with a preference for the acidic or basic cluster respectively. We have computed the frequency distribution for each COG in the acidic, neutral and basic clusters. On analysis, no COG is conserved in the neutral cluster, the largest frequency being 0.5 for COGs 1689, 3016, 3783 and 3801, however their frequency of occurance among all organisms is less than ten percent. This is an expected result as the pI distribution is caused by an interplay of acidic and basic residues which acount for the large acidic and basic clusters. The "neutral" cluster is not caused by the absense or balance of charged residues but because allowed pK combinations of charged residues are minimum at pH 7.1 and 8.4 [5].

Proteins conserved in the acidic and basic clusters however have a preponderance of acidic and basic residues respectively, which maybe required for their function. A complete list of COGs whose protein pI are conserved in these clusters is listed as an additional file. We have scaled the frequency of conservation by the frequency of occurance, so that only COGs which are maximally represented across the organisms in the dataset are used as markers of each cluster. These are listed in Table 2 and Table 3 respectively. The dominant groups of proteins which seem to require an acidic pI are the amino acid tRNA synthetases. Among those proteins whose pI is highly conserved in the basic cluster are a large number of ribosomal proteins. Although highly conserved and intolerant to mutation, ribosomal proteins interweave with negatively charged RNA to form the ribosome, and being positively charged will be a requirement for strong electrostatic interactions.

**Table 2:** COGs conserved in the acidic cluster of the multimodal distribution. P(a) and P(b) are the frequency of occurance in the acidic and basic clusters respectively, F is the function group. The COGs are listed with their P(v) which is score of their variability between the acidic and basic clusters of the pI distribution weighted by their frequency of occurance across all genomes in the dataset. pI values of four organisms – the extremophiles *Buchnera *(environment bacteriocyte), *Halobacterium *(highly acidic – high salt), and *E. coli *and *H. pylori *– intestinal bacteria with different environmental pH.

**COGs with pI conserved in Acidic Cluster**									
**COG**	**P(a)**	**P(b)**	**P(v)**	**F**	**Role**	**Hbs**	**Eco**	**Hpy**	**Buc**

COG2890	0.80	0.18	0.35	J	Predicted rRNA or tRNA methylase	4.23	4.99	5.61	9.38
COG0021	0.81	0.00	0.05	G	Transketolase	-	5.61	6.23	7.98
COG0215	0.82	0.11	0.24	J	Cysteinyl-tRNA synthetase	4.47	5.29	5.88	9.33
COG0016	0.82	0.14	0.32	J	Phenylalanyl-tRNA synthetase alpha subunit	4.14	5.71	6.35	9.51
COG0449	0.82	0.04	0.08	M	Glucosamine 6-phosphate synthetase, contains amidotransferase and phosphosugar isomerase	5.08	5.52	6.05	9.52
COG0206	0.82	0.08	0.16	D	Cell division GTPase	4.35	4.63	5.26	5.08
COG0436	0.82	0.04	0.10	E	PLP-dependent aminotransferases	4.41	6.15	7.41	-
COG0468	0.83	0.15	0.31	L	RecA/RadA recombinase	4.44	5.06	5.47	-
COG0125	0.83	0.17	0.35	F	Thymidylate kinase	4.32	5.26	8.97	10.01
COG0084	0.84	0.03	0.09	L	Mg-dependent DNase	-	5.44	5.43	8.81
COG0504	0.84	0.07	0.18	F	CTP synthase (UTP-ammonia lyase)	4.47	5.58	7.55	9.01
COG0495	0.84	0.11	0.27	J	Leucyl-tRNA synthetase	4.18	5.14	7.51	9.53
COG0124	0.86	0.12	0.27	J	Histidyl-tRNA synthetase	4.29	5.57	5.54	9.28
COG0443	0.86	0.02	0.04	O	Molecular chaperone	3.95	5.08	5.02	7.44
COG0024	0.86	0.08	0.22	J	Methionine aminopeptidase	4.05	5.55	5.89	8.70
COG0209	0.87	0.03	0.10	F	Ribonucleotide reductase alpha subunit	4.39	5.89	5.73	9.11
COG0142	0.87	0.06	0.13	H	Geranylgeranyl pyrophosphate synthase	4.31	4.96	5.94	9.58
COG0441	0.88	0.10	0.23	J	Threonyl-tRNA synthetase	4.35	5.76	5.93	8.86
COG0112	0.88	0.10	0.19	E	Glycine hydroxymethyltransferase	4.30	5.95	6.41	9.22
COG0525	0.88	0.12	0.23	J	Valyl-tRNA synthetase	4.09	5.19	6.12	9.38
COG0060	0.89	0.11	0.23	J	Isoleucyl-tRNA synthetase	4.13	5.63	6.15	9.22
COG0073	0.89	0.08	0.19	R	EMAP domain	4.04	5.29	5.42	8.90
COG0172	0.89	0.09	0.21	J	Seryl-tRNA synthetase	4.60	5.30	6.70	9.42
COG0018	0.89	0.09	0.20	J	Arginyl-tRNA synthetase	4.18	5.29	5.94	9.72
COG0143	0.89	0.09	0.20	J	Methionyl-tRNA synthetase	4.16	5.39	6.00	9.27
COG1109	0.89	0.09	0.21	G	Phosphomannomutase	4.43	5.55	6.18	9.03
COG0006	0.90	0.04	0.11	E	Xaa-Pro aminopeptidase	4.42	5.35	5.73	-
COG0442	0.91	0.09	0.18	J	Prolyl-tRNA synthetase	4.45	5.11	5.90	9.51
COG0126	0.91	0.07	0.14	G	3-phosphoglycerate kinase	4.29	5.06	6.18	9.37
COG0012	0.91	0.09	0.17	R	Predicted GTPase	4.15	4.86	5.50	9.07
COG0149	0.91	0.02	0.09	G	Triosephosphate isomerase	4.15	5.55	7.58	9.23
COG0492	0.92	0.08	0.16	O	Thioredoxin reductase	4.27	5.24	6.02	9.36
COG0072	0.93	0.07	0.14	J	Phenylalanyl-tRNA synthetase beta subunit	4.33	5.26	6.63	8.30
COG0085	0.94	0.02	0.08	K	DNA-directed RNA polymerase beta subunit/140 kD subunit (split gene in Mjan, Mthe, Aful)	4.77	5.14	6.14	7.81
COG0231	0.94	0.02	0.08	J	Translation elongation factor P/translation initiation factor eIF-5A	4.78	5.28	5.34	9.40
COG0459	0.95	0.01	0.04	O	Chaperonin GroEL (HSP60 family)	4.16	4.84	5.51	5.04
COG0013	0.95	0.05	0.09	J	Alanyl-tRNA synthetase	4.29	5.51	5.96	9.42
COG0592	0.96	0.04	0.09	L	DNA polymerase sliding clamp subunit (PCNA homolog)	3.97	5.20	5.45	8.93
COG0202	0.98	0.02	0.05	K	DNA-directed RNA polymerase alpha subunit/40 kD subunit	4.11	4.95	4.95	5.03
COG0148	0.98	0.00	0.00	G	Enolase	4.38	5.28	5.42	6.93
COG0480	0.99	0.01	0.03	J	Translation elongation and release factors (GTPases)	4.41	5.41	5.23	7.61

**Table 3:** COGs conserved in the Basic cluster of the multimodal distribution. Table headers are the same as in Table 2.

**COGs with pI conserved in Basic Cluster**									
**COG**	**P(a)**	**P(b)**	**P(v)**	**F**	**Role**	**Hbs**	**Eco**	**Hpy**	**Buc**

COG0101	0.11	0.82	0.27	J	Pseudouridylate synthase (tRNA psi55)	5.52	8.68	9.74	9.66
COG0582	0.05	0.83	0.12	L	Integrase	5.31	9.70	9.61	-
COG0080	0.15	0.85	0.30	J	Ribosomal protein L11	3.72	9.64	9.55	10.01
COG0477	0.09	0.87	0.20	E	Permeases of the major facilitator superfamily	6.54	9.32	9.14	9.65
COG0477	0.09	0.87	0.20	E	Permeases of the major facilitator superfamily	6.54	9.32	9.14	9.65
COG0477	0.09	0.87	0.20	E	Permeases of the major facilitator superfamily	6.54	9.32	9.14	9.65
COG0477	0.09	0.87	0.20	E	Permeases of the major facilitator superfamily	6.54	9.32	9.14	9.65
COG0541	0.07	0.88	0.19	N	Signal recognition particle GTPase	4.27	9.52	9.40	9.82
COG0255	0.09	0.89	0.21	J	Ribosomal protein L29	4.69	9.98	9.70	10.45
COG0092	0.07	0.93	0.14	J	Ribosomal protein S3	3.87	10.27	10.25	10.40
COG0198	0.07	0.93	0.14	J	Ribosomal protein L24	4.37	10.21	9.86	10.57
COG0051	0.04	0.93	0.11	J	Ribosomal protein S10	5.15	9.68	9.30	9.85
COG0089	0.02	0.95	0.07	J	Ribosomal protein L23	4.13	9.94	10.00	10.00
COG0256	0.05	0.95	0.09	J	Ribosomal protein L18	5.13	10.42	10.16	10.94
COG0087	0.02	0.98	0.05	J	Ribosomal protein L3	5.57	9.90	10.24	10.60
COG0088	0.02	0.98	0.05	J	Ribosomal protein L4	4.83	9.72	9.43	10.19
COG0091	0.02	0.98	0.05	J	Ribosomal protein L22	5.08	10.23	11.26	10.85
COG0098	0.02	0.98	0.05	J	Ribosomal protein S5	4.92	10.11	9.90	10.51
COG0185	0.02	0.98	0.05	J	Ribosomal protein S19	5.20	10.52	10.36	10.73
COG0200	0.02	0.98	0.05	J	Ribosomal protein L15	4.85	11.19	10.52	11.62
COG0201	0.02	0.98	0.05	N	Preprotein translocase subunit SecY	5.56	9.89	9.87	9.72
COG0049	0.02	0.98	0.04	J	Ribosomal protein S7	5.51	10.37	10.17	10.33
COG0081	0.02	0.98	0.04	J	Ribosomal protein L1	4.13	9.64	9.61	9.86
COG0094	0.02	0.98	0.04	J	Ribosomal protein L5	4.66	9.49	9.74	9.89
COG0096	0.02	0.98	0.04	J	Ribosomal protein S8	4.85	9.44	9.79	9.74
COG0097	0.02	0.98	0.04	J	Ribosomal protein L6	4.20	9.71	9.77	10.07
COG0099	0.02	0.98	0.04	J	Ribosomal protein S13	4.52	10.78	10.23	10.54
COG0100	0.02	0.98	0.04	J	Ribosomal protein S11	5.72	11.33	10.33	11.18
COG0102	0.02	0.98	0.04	J	Ribosomal protein L13	4.50	9.91	9.82	9.93
COG0184	0.02	0.98	0.04	J	Ribosomal protein S15P/S13E	4.83	10.40	10.00	10.17
COG0186	0.02	0.98	0.04	J	Ribosomal protein S17	4.75	9.64	10.01	10.14
COG0522	0.02	0.98	0.04	J	Ribosomal protein S4 and related proteins	4.81	10.05	10.08	10.00
COG0199	0.02	0.98	0.04	J	Ribosomal protein S14	5.86	11.16	11.01	11.38
COG0048	0.00	1.00	0.00	J	Ribosomal protein S12	9.91	10.88	10.70	11.09
COG0090	0.00	1.00	0.00	J	Ribosomal protein L2	9.51	10.93	10.36	10.77
COG0093	0.00	1.00	0.00	J	Ribosomal protein L14	9.60	10.43	10.45	10.43
COG0103	0.00	1.00	0.00	J	Ribosomal protein S9	9.39	10.94	10.70	11.40
COG0197	0.00	1.00	0.00	J	Ribosomal protein L16/L10E	9.28	11.23	10.44	10.88

A majority of COGs however show distributions across both the basic and acidic clusters. Knight et al, have shown a correlation to a change in proteome patterns with the organism's ecological niche. Since the proteome always exists in a trimodal distribution, it will only vary from organism to organism in the relative amounts of proteins which are present in each of the three clusters of the pI distribution. We have computed the probability of being in both the acidic and basic clusters weighted by the frequency of occurance in the organisms under consideration in order to identify COGs which are highly represented and show no particular preference for either cluster. This list of COGs, with a joint probability greater than a cutoff value of 0.6 is tabulated in table 4. For reference, the individual values of genes corresponding to the four organisms is also listed, and in a majority of cases show good agreement with the shift in an organism's total theoretical proteome towards either the basic or acidic clusters. Except for some ribosomal proteins, which appear on the list because of their high frequency of occurance, all other COGs are membrane based proteins, which would have direct contact with the external environment. These COGs best represent an organism's shift from expected levels of the acidic and basic clusters of the multimodal distribution, and it is possible that they maybe used as markers to predict an organisms ecological niche, with particular reference to its environmental pH in free living microorganisms.

**Table 4:** Highly occuring COGs with pI varying across both the acidic and basic clusters. Table headers are the same as in Table 2.

**COG**	**P(a)**	**P(b)**	**P(v)**	**F**	**Role**	**Hbs**	**Eco**	**Hpy**	**Buc**
COG0760	0.29	0.31	0.60	O	Parvulin-like peptidyl-prolyl isomerase	-	6.83	9.04	9.65
COG0725	0.31	0.29	0.60	P	ABC-type molybdate transport system, periplasmic component	4.90	7.53	9.70	-
COG0042	0.44	0.25	0.60	R	Predicted TIM-barrel enzymes, possibly dehydrogenases, nifR3 family	4.82	6.04	9.20	-
COG0275	0.28	0.46	0.60	M	Predicted S-adenosylmethionine-dependent methyltransferase	-	5.92	8.99	9.93
COG0258	0.69	0.30	0.61	L	5'-3' exonuclease (including N-terminal domain of PolI)	4.06	5.74	9.06	9.59
COG0640	0.38	0.28	0.61	K	Predicted transcriptional regulators	4.49	8.48	-	-
COG0444	0.37	0.27	0.61	E	ABC-type dipeptide/oligopeptide/nickel transport system, ATPase componen	4.36	6.64	8.29	-
COG0009	0.61	0.30	0.61	J	Putative translation factor (SUA5)	4.43	5.36	-	9.52
COG0226	0.39	0.30	0.62	P	ABC-type phosphate transport system, periplasmic component	4.04	8.37	-	-
COG1475	0.41	0.26	0.62	K	Predicted transcriptional regulators	4.46	6.78	8.39	-
COG0190	0.51	0.30	0.62	H	5,10-methylene-tetrahydrofolate dehydrogenase/Methenyl tetrahydrofolate cyclohydrolase	4.36	5.61	8.74	9.70
COG1057	0.31	0.33	0.62	H	Nicotinic acid mononucleotide adenylyltransferase	-	5.38	8.80	9.68
COG0482	0.42	0.28	0.62	J	Predicted tRNA methyltransferase, contains the PP-loop ATPase domain	-	4.89	8.82	9.50
COG1121	0.29	0.35	0.62	P	ABC-type Mn/Zn transport systems, ATPase component	4.61	9.44	-	9.21
COG0359	0.31	0.48	0.62	J	Ribosomal protein L9	-	5.82	8.80	10.29
COG0858	0.46	0.29	0.62	J	Ribosome-binding factor A	-	5.58	7.55	9.88
COG0356	0.30	0.25	0.62	C	F0F1-type ATP synthase a subunit	-	6.18	6.34	7.64
COG0501	0.29	0.41	0.63	O	Zn-dependent protease with chaperone function	7.18	7.10	7.86	6.91
COG0350	0.31	0.46	0.64	L	Methylated DNA-protein cysteine methyltransferase	5.67	7.13	9.38	-
COG1136	0.53	0.29	0.64	R	ABC-type transport systems, involved in lipoprotein release, ATPase components	6.85	6.66	9.03	7.53
COG0816	0.26	0.36	0.64	L	Predicted endonuclease involved in recombination	-	6.21	9.05	9.81
COG1159	0.36	0.26	0.64	R	GTPases	-	6.54	8.86	9.78
COG0712	0.31	0.29	0.65	C	F0F1-type ATP synthase delta subunit (mitochondrial oligomycin sensitivity protein)	-	4.92	7.56	9.83
COG1160	0.33	0.46	0.65	R	Predicted GTPases	-	5.62	9.15	9.92
COG0463	0.31	0.55	0.66	M	Glycosyltransferases involved in cell wall biogenesis	4.80	8.55	8.87	-
COG0324	0.34	0.30	0.66	J	tRNA delta(2)-isopentenylpyrophosphate transferase	-	5.58	9.56	9.58
COG0617	0.32	0.36	0.66	J	tRNA nucleotidyltransferase/poly(A) polymerase	-	7.84	9.14	9.35
COG1385	0.35	0.32	0.67	S	Uncharacterized BCR	-	6.80	9.08	9.71
COG1825	0.34	0.34	0.67	J	Ribosomal protein L25 (general stress protein Ctc)	-	9.60	9.41	9.91
COG0358	0.63	0.30	0.67	L	DNA primase (bacterial type)	4.35	5.62	8.97	9.48
COG0593	0.42	0.32	0.69	L	ATPase involved in DNA replication initiation	-	6.97	8.35	9.55
COG0052	0.64	0.33	0.69	J	Ribosomal protein S2	4.06	6.38	6.82	9.61
COG0616	0.49	0.32	0.69	N	Periplasmic serine proteases (ClpP class)	4.93	7.44	9.41	9.66
COG0212	0.39	0.35	0.70	H	5-formyltetrahydrofolate cyclo-ligase	4.42	6.10	9.98	-
COG0470	0.59	0.34	0.70	L	ATPase involved in DNA replication	4.47	6.46	8.70	9.39
COG1132	0.33	0.44	0.70	Q	ABC-type multidrug/protein/lipid transport system, ATPase component	4.43	6.91	9.23	9.60
COG0750	0.33	0.52	0.71	M	Predicted membrane-associated Zn-dependent proteases 1	4.91	6.41	9.18	-
COG0341	0.36	0.40	0.71	N	Preprotein translocase subunit SecF	4.53	5.46	8.73	-
COG0475	0.35	0.32	0.71	P	Kef-type K+ transport systems, membrane components	-	5.13	9.12	-
COG0566	0.43	0.35	0.72	J	rRNA methylases	-	7.15	9.18	-
COG0438	0.35	0.46	0.73	M	Predicted glycosyltransferases	4.96	8.21	8.66	-
COG0223	0.43	0.36	0.73	J	Methionyl-tRNA formyltransferase	-	6.01	8.87	9.80
COG0237	0.60	0.36	0.74	H	Dephospho-CoA kinase 1	4.59	5.64	8.91	10.26
COG0668	0.36	0.42	0.76	M	Small-conductance mechanosensitive channel	5.29	7.56	8.87	9.50
COG0532	0.57	0.34	0.77	J	Translation initiation factor 2 (GTPase)	4.33	5.76	6.93	9.38
COG0484	0.35	0.41	0.82	O	Molecular chaperones (contain C-terminal Zn finger domain)	4.54	7.94	8.12	9.17
COG0164	0.39	0.44	0.83	L	Ribonuclease H	4.41	6.91	8.95	-
COG0020	0.38	0.47	0.85	I	Undecaprenyl pyrophosphate synthase	5.11	6.29	8.97	9.45
COG0681	0.41	0.49	0.86	N	Signal peptidase	4.99	6.46	8.34	9.51
COG0244	0.40	0.53	0.87	J	Ribosomal protein L10	3.80	9.04	9.36	9.98
COG0130	0.45	0.43	0.88	J	Pseudouridine synthase	5.37	5.59	-	9.74
					**Mean**	4.76	6.64	8.75	9.47
					**Standard Deviation**	0.7	1.14	0.73	0.69

Extrapolating results obtained from using the theoretical proteome must be viewed with caution as predicted pI values are for unfolded proteins obtained from sequence and not the native folded proteins in the cellular microenvironment, which remain unknown. We have resisted attempting to correlate observations of an ortholog's thoeretical pI with its function, unless clearly obvious, for this reason. The theoretical proteome is also generated from the total set of proteins present in an organism's genome, while only a subset maybe expressed at any given time and will vary in response to external stimuli. An organism may also respond to evolutionary pressure such as environmental pH by increasing the number of copies of a charged protein, a ploy frequently adopted in drug resistance. The effect of a shift in the relative levels of the acidic and basic peaks maybe replicated by an increase in copy number of proteins belonging to the relevant cluster. As theoretical proteome studies are not weighted by the copy number of the individual proteins, for lack of relevant data related to the quantity of individual proteins for the entire proteome, these observations are impossible to make.

## Conclusion

Analysis of ortholog pI across forty two microorganism genomes which contain reprentatives of free living archea and bacteria are analysed to identify orthologs which are invariant in pI as well as those amenable to changes in pI. Orthologs with a high frequency of occurance and variation in pI are shortlisted, and maybe used as markers in future studies which attempt to map proteome properties to variation in the organisms ecological niche.

## Methods

Genome sequences and the COG database generated with 43 organisms with sequences and alignments were sourced from NCBI [15]. *Sacharomyces cerevisae *was removed from the dataset so that it contained only archeae and bacteria which were single-celled organisms. This was intentionally to include only genomes of organisms which would have their membrane directly in contact with the external environment and included a range of organisms with diverse ecological niches.

The isoelectric point is determined from an equation subtracting the sum of all negative charges (of all acidic residues) from the sum of all positive charges (of all basic residues), by varying the pH by bisectional nesting of intervals on the pH scale until the interval is smaller than a given level (0.001), as described in [2]. Fixed values for pK are considered to calculate the isoelectric point, and are the same as used in the molecular weight/isoelectric point program at [16].

The molecular weights and isoelectric point of all the predicted open reading frames from the completed genomes of a number of prokaryotes were generated. All ORFs that are classified by respective COG identities and assigned to one of the 18 functional groups [12] were used in comparitive analysis between genomes.

Correlation of molecular weights and pI were performed, using scripts written in house, for all COGs classified to a particular function for each pair of organisms. Pairwise distances were calculated for all pairs of proteins belonging to the specified COG. Sequences assigned to each COG were aligned with ClustalW [17] using default parameters, and the resulting multiple alignment used to generate a distance matrix with the program PROTDIST, using default parameters (Jones-Taylor-Thornton model, where the distance is scaled in units of the expected fraction of amino acids changed), from the Phylip package [18]. Perl scripts used to automate this protocol were written using BioPerl Modules [19].

Individual molecular weight and isoelectric point along with pairwise distances were stored in a MySQL database v 4.1.9, running on a Fedora Core Linux dual Xeon Server, to enable easy retrieval. Statistical properties on the data (mean, variance and correlation coefficients) were calculated using standard methods after sectioning the data at the levels of COGs and functional groups. Figures plotted in this paper were made using Sigmaplot (Systat Software Inc, Richmond, California, USA).

Statistical significance of functional groups was assessed using non-parametric Kruskal-Wallis tests and Dunn's Multiple Comparison [20] tests (alpha = 0.2), after randomly sampling three hundred sequences from each group, and from the total population to form the sample sets.

To score the COGs both for variation and conservation, probabilities were estimated from frequencies in the dataset. The frequency of occurance of a COG *i, F(i)*, calculated as



Where n_o _is the number of organisms in which the COG is present and n_t _is the number of organisms in the sample dataset (42).



where *i *is the cog, *j *is the cluster – either acidic, neutral or basic, and *r *the range corresponding to the cluster – 0–7.4 for acidic, 7.4–8.1 for basic, and 7.4–14 for basic. n_r _is the number of proteins in the given cluster, n_i _total number of proteins corresponding to *i*. P_i _(j) is the conservation score for the cluster j, and is the joint probabiity of conservation and occurance of the COG *i*.

We estimate the joint probability of variation between the acidic and basic clusters for a COG *i*, P_i_(v), as the probability of variation into its frequency of occurance calculated as



where n_a _and n_b _are the number of proteins belonging to the COG *i *in the acidic and basic cluster of the distribution respectively, n_i _being the total number of proteins classified under COG *i *.

Membrane proteins were predicted using TOPPRED [21].

## Authors' contributions

Mr Soumyadeep Nandi did all computation and database work, Mr. Nipun Mehra started this work, and generated preliminary results, Dr. Andrew Lynn guided the implementation of the project and wrote the manuscript, Prof. Alok Bhattacharya provided overall guidance and interpretation of results.

## Supplementary Material

Additional file 1 **List of COGS sorted by mean pI. **File with rows containing COG, mean, standard deviation and pI of individual ortholog proteins that were used in the calculation described in this paper. The rows are sorted by mean pI (column 2) and used to plot figure 5 of the manuscript. The file is tab-delimited and will open in any spreadsheet program. Click here for file

Additional file 2 **COGs with pI conserved in acid and basic clusters. **Text file containing the comprehensive list of COGs which are conserved in either the acidic or basic cluster of the multimodal distribution. Click here for file
